# Astrocytes in the Ventromedial Hypothalamus Involve Chronic Stress-Induced Anxiety and Bone Loss in Mice

**DOI:** 10.1155/2021/7806370

**Published:** 2021-07-08

**Authors:** Yunhui Liu, Jie Shao, Dashuang Gao, Lu Zhang, Fan Yang

**Affiliations:** ^1^The Brain Cognition and Brain Disease Institute, Shenzhen Institute of Advanced Technology, Chinese Academy of Sciences, Shenzhen-Hong Kong Institute of Brain Science-Shenzhen Fundamental Research Institutions, Shenzhen 518055, China; ^2^University of Chinese Academy of Sciences, Beijing 100049, China

## Abstract

Chronic stress is one of the main risk factors of bone loss. While the neurons and neural circuits of the ventromedial hypothalamus (VMH) mediate bone loss induced by chronic stress, the detailed intrinsic mechanisms within the VMH nucleus still need to be explored. Astrocytes in brain regions play important roles in the regulation of metabolism and anxiety-like behavior through interactions with surrounding neurons. However, whether astrocytes in the VMH affect neuronal activity and therefore regulate chronic stress-induced anxiety and bone loss remain elusive. In this study, we found that VMH astrocytes were activated during chronic stress-induced anxiety and bone loss. Pharmacogenetic activation of the Gi and Gq pathways in VMH astrocytes reduced and increased the levels of anxiety and bone loss, respectively. Furthermore, activation of VMH astrocytes by optogenetics induced depolarization in neighboring steroidogenic factor-1 (SF-1) neurons, which was diminished by administration of N-methyl-D-aspartic acid (NMDA) receptor blocker but not by alpha-amino-3-hydroxy-5-methyl-4-isoxazolepropionic acid (AMPA) receptor blocker. These results suggest that there may be a functional “glial-neuron microcircuit” in VMH nuclei that mediates anxiety and bone loss induced by chronic stress. This study not only advances our understanding of glial cell function but also provides a potential intervention target for chronic stress-induced anxiety and bone loss therapy.

## 1. Introduction

Chronic stress can lead to different mental disorders, manifesting as anxiety, depression, panic, and other symptoms [1–3]. Importantly, chronic stress-induced anxiety often causes a variety of metabolic problems, including glucose [4, 5], lipid [6, 7], and bone metabolism disorders [8, 9]. In particular, abnormal bone metabolism is a common symptom of stress and anxiety [10, 11]. Clinical studies have shown that the probability of osteoporosis and fractures in patients with anxiety and depression is significantly higher than that in normal controls [12–14], suggesting that an individual's anxiety state is closely related to bone metabolism. Although evidence indicates that anxiety can affect bone loss, the underlying neural mechanism is still unclear.

The VMH is closely related to mood disorders such as anxiety and depression. Studies have shown that VMH brain activity is significantly increased in cats with anxiety-like symptoms [15, 16], while blocking VMH glutamate signals can effectively reduce anxiety in animals [17], suggesting that the VMH is involved in the regulation of mood disorders. Furthermore, cannabinoid [18, 19] and serotonin receptors [19, 20] in steroidogenic factor-1- (SF-1-) positive neurons, the main neuronal subtype in the VMH, mediate regulation of anxiety behavior in mice. Research has also shown that bone mass increases significantly after chemical damage to SF-1 neurons in the VMH, suggesting a role of VMH in the regulation of bone metabolism [21]. Subsequent studies have demonstrated that leptin and serotonin act on SF-1 neurons to regulate bone metabolism by regulating sympathetic nerve activity [22–25]. Thus, SF-1 neurons in the VMH are crucial for central regulation of anxiety and bone metabolism. We previously revealed a BNST^SOM^-VMH^SF-1^-NTS^Vglut2^ neural circuit that regulates the activities of the peripheral sympathetic nervous system and mediates bone loss caused by chronic stress [26]. As a key link in this circuit, the VMH is responsible for integrating “anxiety information” sent by the upstream bed nucleus of the stria terminalis (BNST) and “bone regulation information” from the downstream—the nucleus of the solitary tract (NTS). To date, however, the underlying neural mechanism within the VMH nucleus remains uncertain regarding regulation of chronic stress-induced anxiety and bone loss.

Astrocytes, which are the most abundant cell type in the brain, are considered to play an auxiliary and supportive role in advanced cognitive functions in the brain. However, increasing evidence suggests that astrocytes are directly involved in the mediation of advanced emotions and metabolic regulation [27–30]. Astrocytes in the lateral habenula (LHb) regulate depressive behavior in rats via the Kir4.1 potassium channel, which is selectively expressed in astrocytes [27]. Hippocampal astrocytes regulate major depression in mice via astrocyte-derived adenosine triphosphate (ATP) [28], suggesting an important role in the regulation of negative emotions such as anxiety and depression. Importantly, hypothalamic VMH astrocytes regulate activities of hypothalamic agouti-related peptide (AgRP) and proopiomelanocortin (POMC) neurons through cannabinoid (CBR1) and adenosine receptors and thereby regulate feeding and metabolism in mice [29, 30]. Thus, given the important role of astrocytes in anxiety and metabolic regulation, we hypothesized that VMH astrocytes likely play a critical role in the regulation of bone loss induced by chronic stress.

In this study, we achieved bidirectional regulation of chronic stress-induced anxiety and bone loss in mice by the chemogenetic manipulation of VMH astrocytes. Specifically, activation of the Gi pathway in VMH astrocytes prevented chronic stress-induced anxiety-like behavior and bone loss, whereas activation of the Gq pathway exerted the opposite effects. Furthermore, the neuronal electrophysiological recordings suggested that these effects may be mediated by astrocyte to SF1 neuron signaling via NMDA receptors but not AMPA receptors. Collectively, this study indicated that VMH astrocytes not only participate in the regulation of mood disorders but can intervene in bone metabolism, thus providing a potential interventional target for the therapeutic treatment of chronic stress-induced anxiety and bone loss.

## 2. Results

### 2.1. Chronic Stress-Induced Anxiety and Bone Loss Promote c-fos Expression in VMH Nuclei

**Figure 1:**
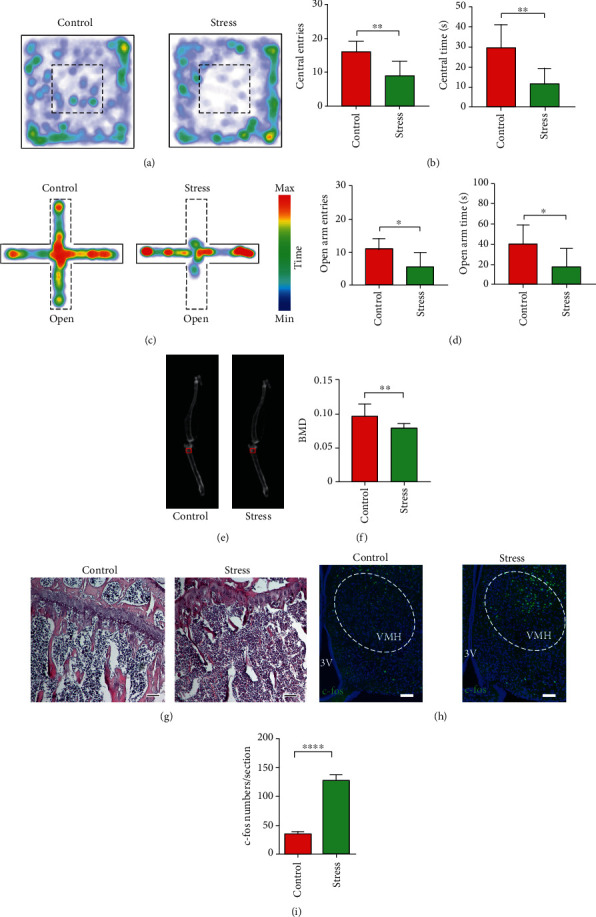
Chronic stress induced anxiety-like behavior and bone loss in mice. (a) Representative heat maps of control and stress groups in different positions in open field test (OFT), with warmer color indicating more time spent at that location. (b) Quantification of entries into and time spent in central area in control and stress groups; values represent mean ± SD (*n* = 8 for control and *n* = 7 for stressed group; ^∗∗^*p* < 0.01; unpaired *t*-test). (c) Representative heat maps of control and stress groups in different positions in elevated plus maze test (EPM), with warmer color indicating more time spent at that location. (d) Quantification of entries into and time spent in open arm in control and stress groups; values represent mean ± SD (*n* = 8 for control and *n* = 7 for stress group; ^∗^*p* < 0.05; unpaired *t*-test). (e) Representative dual-energy X-ray image showing bone mineral density (BMD) of control and stress groups. BMD in red box was collected for statistical analysis. (f) Quantification of BMD in control and stress groups; values represent mean ± SD (*n* = 10 per group from 5 mice per group; ^∗∗^*p* < 0.01; unpaired *t-*test). (g) Representative hematoxylin and eosin (H&E) staining of proximal tibia in control and stress groups. Number of proximal tibia trabeculae in stressed mice is less than in control mice; scale bar, 100 *μ*m. (h) c-fos staining of VMH in control and stress groups; scale bar, 100 *μ*m. (i) Quantification of c-fos-positive cells in control and stress groups; values represent mean ± SD (*n* = 20 sections from 5 mice per group; ^∗∗∗∗^*p* < 0.0001; unpaired *t*-test).

We previously identified a BNST-VMH-NTS neural circuit that mediates chronic stress-induced bone loss, whereby the VMH integrates “anxiety information” from the BNST and “bone regulation information” from the NTS [26]. To investigate the role of the VMH in chronic stress-induced anxiety and bone loss, we established a chronic unpredictable mild stress-induced anxiety model in mice. After 8 weeks of chronic stress, mice displayed a decrease in the frequency of entries and time spent in the central area in the open field test compared with the control mice (Figures 1(a) and 1(b)). In addition, compared with the control mice, stressed mice entered less often and spent significantly less time in the open arms of the elevated plus maze (Figures 1(c) and 1(d)). These results indicate that chronic stress (8 weeks) can induce anxiety-like behavior in mice. Importantly, dual-energy X-ray scanning analysis showed that bone density of the proximal tibia was significantly lower in stressed mice than in control mice (Figures 1(e) and 1(f)). Furthermore, H&E staining also indicated a significantly lower number of proximal tibia trabeculae in the stressed group than in the control group (Figure 1(g)). Based on immunohistochemical staining, expression of c-fos in the VMH brain area of the stressed group was significantly higher than that in the control group (Figures 1(h) and 1(i)), suggesting that neural activity in the VMH nucleus is involved in the maintenance of the anxiety state and decrease in bone mineral density (BMD).

### 2.2. Activation of VMH Astrocytes in Chronic Stress-Induced Anxiety and Bone Loss

**Figure 2:**
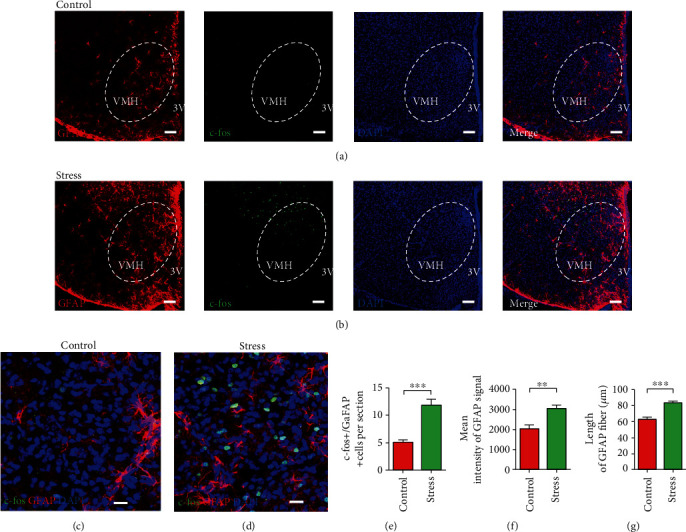
Astrocytes in VMH are activated in chronic stress-treated mice. (a) Representative low magnification image showing triple staining of GFAP (red), c-fos (green), and DAPI (blue) in VMH of control group; scale bar, 100 *μ*m. (b) Representative low magnification image showing triple staining of GFAP (red), c-fos (green), and DAPI (blue) in VMH of stress group; scale bar, 100 *μ*m. (c) Representative high magnification image showing triple staining of GFAP (red), c-fos (green), and DAPI (blue) in VMH of control group; scale bar, 20 *μ*m. (d) Representative high magnification image showing triple staining of GFAP (red), c-fos (green), and DAPI (blue) in VMH of stress group; scale bar, 20 *μ*m. (e) Quantification of c-fos^+^ and GFAP^+^ coexpressed cells in control and stress groups; values represent mean ± SD (*n* = 20 sections from 5 mice per group; ^∗∗∗^*p* < 0.001; unpaired *t*-test). (f) Mean signal intensities of GFAP staining in VMH of control and stress groups; values represent mean ± SD (*n* = 5 mice per group; ^∗∗^*p* < 0.01; unpaired *t*-test). (g) The length of GFAP fiber in VMH astrocytes of control and stress groups; values represent mean ± SD (*n* = 20 cells; 4 cells from each mouse, 5 mice per group; ^∗∗∗^*p* < 0.001; unpaired *t*-test).

Astrocytes are involved in the regulation of emotional disorders and metabolic functions [24–27]. Thus, we examined the role of astrocytes in the VMH nucleus in chronic stress-induced anxiety and bone loss. Based on immunostaining, the number of c-fos and glial fibrillary acidic protein (GFAP) coexpressing astrocytes in the VMH of stressed mice increased significantly compared with that in the control group (Figures 2(a)–2(e)). These results suggest that VMH astrocyte activity is closely related to maintenance of the anxiety state and decreased BMD. In addition, increased GFAP signals and GFAP fibers were observed in the VMH astrocytes of the stress group compared with that of control mice, suggesting alteration in the structure of astrocytes after chronic stress (Figures 2(f) and 2(g)). Therefore, we hypothesized that chronic stress impacts VMH astrocyte activity to regulate the activities of SF-1 neurons in the VMH and thereby modulating the process of bone loss induced by chronic stress.

### 2.3. Activation of Gi Pathway in VMH Astrocytes Reduces Anxiety and Prevents Bone Loss

**Figure 3:**
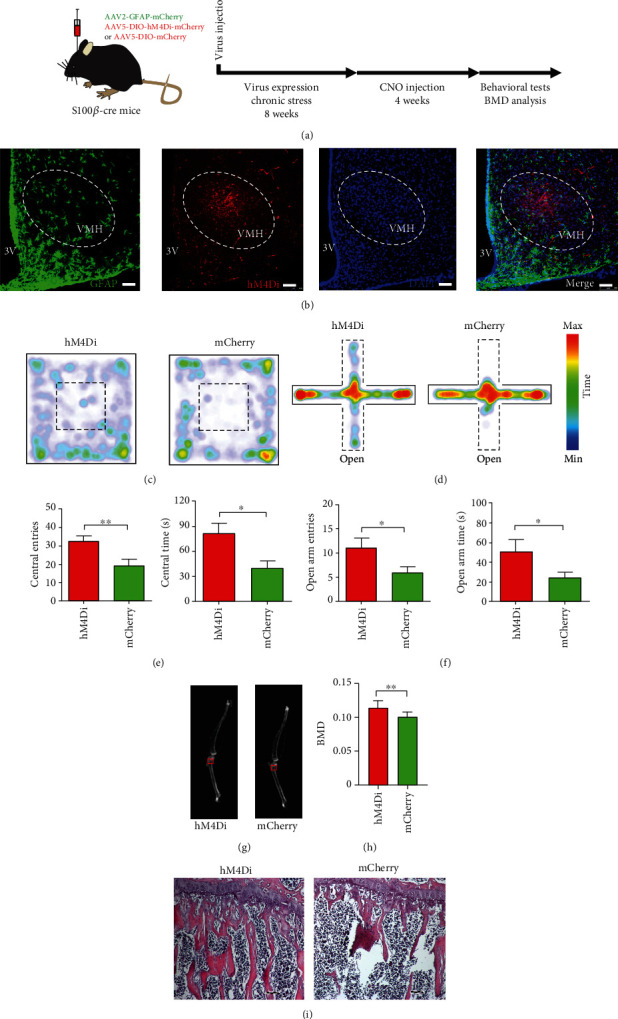
Activation of Gi pathway in VMH astrocytes decreases anxiety-like behavior and prevents bone loss. (a) Schematic of experimental procedure for mice with chronic stress and chemogenetic inhibition. (b) Representative image showing GFAP and hM4Di virus expression in VMH of S100B-Cre mouse (green, GFAP; red, hM4Di-mCherry; blue, DAPI; scale bars, 60 *μ*m). (c) Representative heat maps of hM4Di and mCherry mice in different positions in OFT. (d) Representative heat maps of hM4Di and mCherry mice in different positions in EPM. (e) Quantification of entries into and time spent in central area in hM4Di and mCherry groups; values represent mean ± SD (*n* = 10 for hM4Di and *n* = 8 for mCherry group; ^∗^*p* < 0.05; ^∗∗^*p* < 0.01; unpaired *t*-test). (f) Quantification of entries into and time spent in open arm in hM4Di and mCherry groups; values represent mean ± SD (*n* = 10 for hM4Di and *n* = 8 for mCherry group; ^∗^*p* < 0.05; unpaired *t*-test). (g) Representative dual-energy X-ray image showing BMD in hM4Di and mCherry groups. BMD in red box was collected for statistical analysis. (h) Quantification of BMD in hM4Di and mCherry groups; values represent mean ± SD (*n* = 14 from 7 hM4Di mice and *n* = 10 from 5 mCherry mice; ^∗∗^*p* < 0.01; unpaired *t*-test). (i) Representative images of H&E staining showing chemogenetic inhibition of VMH astrocytes rescued the decrease in proximal tibia trabeculae caused by chronic stress (scale bar, 100 *μ*m).

To test the hypothesis that VMH astrocyte activity mediates chronic stress-induced anxiety-like behavior and decreases the BMD, we injected AAV2-DIO-hM4Di-mCherry or AAV2-DIO-mCherry into the VMH of S100*β*-Cre mice, which enabled the selective activation of the Gi pathway in VMH astrocytes by clozapine N-oxide (CNO) administration (Figures 3(a) and 3(b)). After 4 weeks of viral expression and 8 weeks of daily stress, mice received an intraperitoneal injection of CNO (1 mg/kg) to investigate the behavioral effects of Gi pathway activation in VMH astrocytes (Figure 3(a)). Results showed that selective activation of the Gi pathway in VMH astrocytes effectively prevented the induction of anxiety-like behavior following chronic stress. Compared with the control group mice, the hM4Di-expressing mice entered more frequently and spent more time in the central area of the open field (Figures 3(c) and 3(e)). In addition, compared with the control group, the hM4Di-expressing mice preferred to enter and stay in the open arms of the elevated plus maze (Figures 3(d) and 3(f)). To determine the effect of Gi pathway activation in VMH astrocytes on the BMD of mice, we injected CNO into mice three times a week for 4 weeks. Dual energy X-ray bone scanning showed that the BMD of the proximal tibia of the hM4Di group was significantly higher than that of the control group (Figures 3(g) and 3(h)). Furthermore, the number of proximal tibia trabeculae in the hM4Di group was significantly greater than that in the mCherry-expressing control group (Figure 3(i)). In summary, selective activation of the Gi pathway in VMH astrocytes effectively reduced anxiety-like behavior and prevented BMD decrease induced by chronic stress.

### 2.4. Activation of Gq Pathway in VMH Astrocytes Induces Anxiety and Promotes Bone Loss

**Figure 4:**
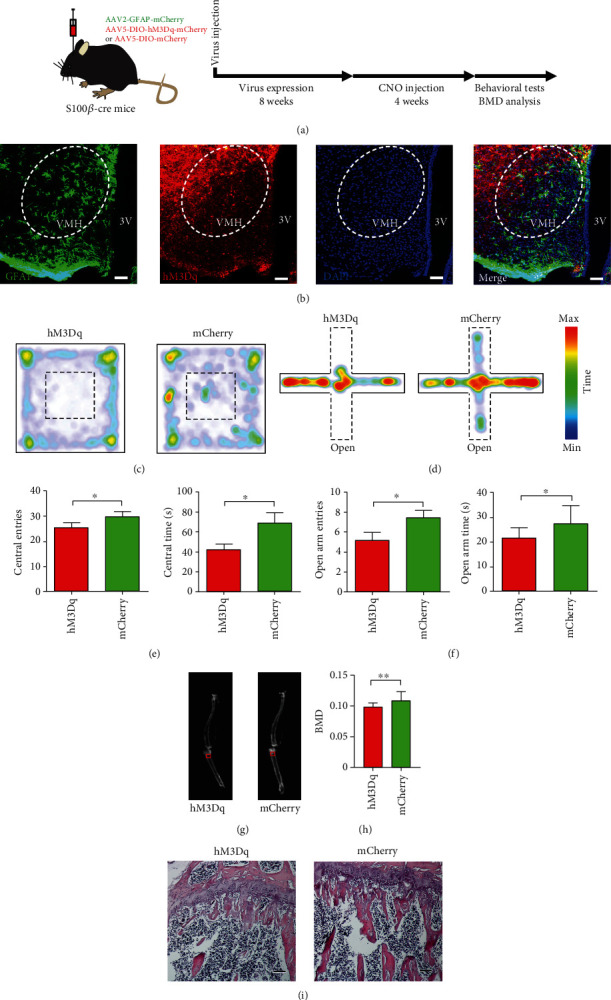
Activation of Gq pathway in VMH astrocytes increases anxiety-like behavior and promotes bone loss. (a) Schematic of experimental procedure for mice with chemogenetic activation. (b) Representative image showing GFAP and hM3Dq virus expression in VMH of S100B-Cre mouse (green, GFAP; red, hM3Dq-mCherry; blue, DAPI; scale bars, 60 *μ*m). (c) Representative heat maps of hM3Dq and mCherry mice in different positions in OFT. (d) Representative heat maps of hM3Dq and mCherry mice in different positions in EPM. (e) Quantification of entries into and time spent in central area in hM3Dq and mCherry groups; values represent mean ± SD (*n* = 10 for hM3Dq and *n* = 10 for mCherry group; ^∗^*p* < 0.05; ^∗∗^*p* < 0.01; unpaired *t*-test). (f) Quantification of entries into and time spent in open arm in hM3Dq and mCherry groups; values represent mean ± SD (*n* = 10 for hM4Di and *n* = 10 for mCherry group; ^∗^*p* < 0.05; unpaired *t*-test). (g) Representative dual-energy X-ray image showing BMD of hM3Dq and mCherry groups. BMD in red box was collected for statistical analysis. (h) Quantification of BMD in hM3Dq and mCherry groups; values represent mean ± SD (*n* = 14 from 7 hM3Dq mice and *n* = 16 from 8 mCherry mice; ^∗∗^*p* < 0.01; unpaired *t*-test). (i) Representative image of H&E staining showing that chemogenetic activation of VMH astrocytes mimicked the decrease in proximal tibia trabeculae caused by chronic stress (scale bar, 100 *μ*m).

We next explored whether the Gq pathway in VMH astrocytes also participates in the regulation of anxiety-induced bone loss. First, we selectively expressed hM3Dq or mCherry in the astrocytes of the VMH nucleus by stereotactic injection in S100*β*-Cre mice (Figures 4(a) and 4(b)). Following intraperitoneal injection of CNO (1 mg/kg), Gq pathway activation in the VMH astrocytes induced obvious anxiety-like behavior in mice. The number of entries and time spent exploring the central area of the open field was significantly lower in the hM3Dq-expressing mice than in the control mice (Figures 4(c) and 4(e)). Consistently, compared with the control group, hM3Dq-expressing mice entered less often and spent significantly less time in the open arms of the elevated plus maze (Figures 4(d) and 4(f)). Notably, dual energy X-ray analysis showed that BMD of the proximal tibia was significantly lower in the hM3Dq-expressing mice than in the control group (Figures 4(g) and 4(h)). The H&E staining results showed that the number of proximal tibia trabeculae was significantly lower in the hM3Dq-expressing group than in the mCherry-expressing control group (Figure 4(i)). These results suggest that activation of the Gq pathway in VMH astrocytes can induce significant anxiety-like behavior and promote BMD decrease in mice. Thus, pharmacogenetic activation of the Gi and Gq pathways in VMH astrocytes can bidirectionally regulate anxiety-like behavior and bone loss in mice.

### 2.5. Optical Stimulation of Astrocytes Induced an Excitatory Response in SF-1 Neurons via NMDA Receptor

**Figure 5:**
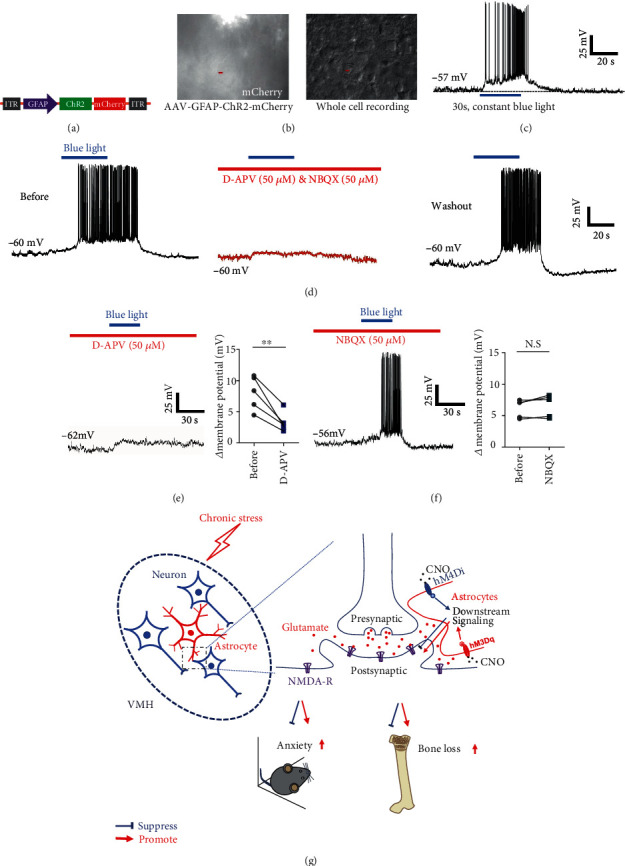
Optogenetically activated astrocytes induced excitatory response in SF-1 neurons, mainly via NMDA receptor. (a) Schematic representing viral construct of ChR2 specifically expressed in astrocytes. (b) Whole-cell recording of VMH neurons near ChR2-expressing astrocytes. (c) Representative recordings illustrating VMH SF-1 neurons depolarized by optical stimulation of astrocytes. (d) Application of glutamatergic receptor antagonists NBQX (50 *μ*M) and D-APV (50 *μ*M) suppressed action potential firing caused by optical stimulation of astrocytes, which was recovered after aCSF washout (*n* = 6). (e) Application of D-APV significantly diminished membrane potential depolarization induced by astrocyte activation (*n* = 5, ^∗∗^*p* < 0.01; paired *t*-test). (f) NBQX did not influence interaction between astrocytes and SF-1 neurons (*n* = 5, ^∗^*p* < 0.05; paired *t*-test). (g) Schematic showing that manipulation of astrocytes in VMH could bidirectionally regulate chronic stress-induced anxiety and bone loss by affecting neural excitatory through NMDA receptors.

To clarify the neural mechanism underlying VMH astrocyte regulation of anxiety-like behavior and bone metabolism, we applied optogenetics to test the effects of astrocyte activation on SF-1 neurons, which are crucial regulators of anxiety and bone metabolism [21, 25, 31]. By injecting AAV-GFAP-ChR2-mCherry into the VMH of mice, we achieved optical activation of astrocytes in brain slices during patch recordings (Figures 5(a) and 5(b)). Results indicated that optical stimulation of astrocytes depolarized SF-1 neurons and induced action potential firing (Figure 5(c)). As glutamate is considered a primary excitatory neurotransmitter, we applied glutamate receptor antagonists to brain slices to explore the interactions between astrocytes and SF-1 neurons. Among glutamate receptors, ionotropic glutamate receptors (iGluRs), including NMDA, AMPA, and kainate receptors, can mediate rapid depolarization by glutamate [32]. Given that NBQX and D-APV can block most iGluRs, we added these antagonists to perfused artificial cerebrospinal fluid (aCSF) and found that action potential firing of SF-1 neurons caused by optical activation of astrocytes was significantly suppressed (Figure 5(d)), suggesting that depolarization was primarily due to activation of glutamate receptors. Therefore, we applied NBQX and D-APV separately into aCSF to clarify the main glutamate receptor subtype involved in astrocyte activation-induced SF-1 neuronal firing. As shown in Figure 5, application of D-APV, but not NBQX, significantly blocked SF-1 neuronal firing caused by optical activation of astrocytes. We also recorded changes in membrane potential in SF-1 neurons before and after the application of blue light and found that D-APV markedly diminished the membrane potential depolarization induced by astrocyte activation, while NBQX exerted no obvious effect (Figures 5(e) and 5(f)). Taken together, we concluded that astrocytes regulate SF-1 neuronal activity, which, in turn, regulates anxiety-like behavior and bone loss via NMDA receptors.

## 3. Discussion

Stress-induced emotional disorders, such as anxiety and depression, are closely related to bone loss [8, 11–13]. Denes and colleagues identified fluorescent signals in several brain regions following injection of pseudorabies virus (PRV) into the bone marrow cavity of rats, thereby proving a physical neural connection between bone and brain [33]. These findings suggest that the central nervous system may be involved in the regulation of bone metabolism. The hypothalamus is a crucial brain area for regulating visceral and endocrine activities, which not only regulates body temperature [34], food intake [35], energy metabolism [36], and other basic functions closely related to biological survival but also participates in animal emotion and biological rhythm regulation [37, 38]. The VMH plays a crucial role in energy metabolism [39], bone metabolism [22, 24], and emotional regulation [15, 16]. We previously identified a BNST-VMH-NTS neural circuit mediating chronic stress-induced anxiety and bone loss [26] and revealed the regulatory mechanism of anxiety-induced bone loss at the central circuit level. However, the mechanism related to chronic stress-induced anxiety and bone loss regulation by the VMH nucleus as an intermediate linker of the neural circuit remains unclarified.

Using DREADD (designer receptors exclusively activated by designer drugs) manipulation, we found that selective activation of the Gi pathway in the VMH astrocytes prevented chronic stress-induced anxiety-like behavior and bone loss, whereas activation of the Gq pathway in VMH astrocytes induced anxiety-like behavior and bone loss in normal wild-type mice. Combining optogenetics and electrophysiological recordings, we found that selective activation of VMH astrocytes induced an excitatory response in SF-1 neurons, which was mediated through the NMDA receptor. Although our results cannot confirm the mechanism by which astrocytes regulate the NMDA receptor of SF-1 neurons, astrocytes may potentially induce an excitatory response in SF-1 neurons by enhancing the uptake of GABA or secreting more glial-derived glutamate. Based on the ability of astrocytes to regulate glutamate concentration in the extracellular space and elevate extrasynaptic NMDA receptor expression and/or activation in disease states [40, 41], we hypothesized that photoactivation of astrocytes may excite SF-1 neurons via extrasynaptic NMDA receptors. Furthermore, our results also showed enhanced GFAP signals in the VMH after chronic stress. Altered expression of GFAP is reported to affect extrasynaptic gliotransmission due to the interaction between scaffold protein GFAP and transporters on the membrane, thus affecting neuronal activity [42].

### 3.1. Regulation of Astrocyte Activity by Chemogenetics and Optogenetics

Astrocytes, a major glial cell type in the brain, respond to neuronal activities by increasing intracellular calcium events, which, in turn, trigger gliotransmitter secretion and neural activities [43–45]. Astrocytes also modulate the excitability of neurons through K^+^ clearance via the potassium channel Kir4.1, which changes the concentration of potassium ions in the extracellular environment [46]. Kir4.1, which is upregulated in LHb astrocytes in depressed rats, tightly regulates the degree of membrane hyperpolarization and amount of bursting activity in LHb neurons, and astrocyte-specific gain and loss of Kir4.1 in the LHb bidirectionally regulates neuronal bursting and depressive-like symptoms [27]. By secreting diverse gliotransmitters, astrocytes are also involved in regulation of behavior in mice [28, 47]. Astrocytes are capable of expressing virtually all types of neurotransmitter receptors. These receptors can be activated by synaptically released neurotransmitters, which makes astrocytes indispensable for many physical functions [48–50].

Optogenetics have been widely used to control neuronal activities [51], as well as to regulate the activities and functions of cells other than neurons, including islet cells [52–54], cardiomyocytes [55, 56], and astrocytes [57, 58]. Due to the regulation effects of astrocytes on neuronal activity, considerable effort has been made to study the optogenetic regulation of astrocytes, including on neuronal activity, neural circuit function, and animal behavior [27, 59, 60]. Pharmacogenetics (e.g., DREADD), also known as chemogenetics, can be applied to express artificially constructed receptor channels on specific cells and manipulate receptor channels through artificial application of specific ligand CNO to affect cellular activity [57]. Pharmacogenetics can also change the concentration of intracellular calcium ions and the activity of intracellular kinase, thus influencing cell activity regulation [61]. The most widely used DREADD elements include mutated human muscarinic receptors hM3Dq and hM4Di. hM3Dq can activate the intracellular Gq signaling pathway for excitatory stimulation of neurons, while hM4Di can activate the intracellular Gi pathway for inhibitory stimulation of neurons. Both are widely used in mice and nonhuman primates [62, 63].

Compared with highly spatiotemporal-specific optogenetic regulation technology, chemogenetics usually takes about 30 min to work, so time specificity is weaker than that of optogenetics. However, because long-term chronic stimulation is not a real-time response, and intraperitoneal injection of CNO is noninvasive, it is often used in chronic behavioral research: Adamsky and colleagues applied DREADD technology to selectively activate the Gq pathway in astrocytes from the hippocampal CA1 subregion, which enhanced the formation of short-term memory in mice [64]. However, they also found that chemogenetic activation of the Gi pathway in astrocytes from the hippocampal CA1 region prevents long-term memory in mice [65]. These studies indicate that chemogenetic manipulation of astrocytes is a feasible approach for studying their physiological functions.

By delivering the AAV-DIO virus into the VMH of S100*β*-Cre mice, we specifically expressed hM3Dq or hM4Di in the astrocytes of the VMH nucleus. After intraperitoneal injection of CNO, we found that chemogenetic regulation of VMH astrocytes bidirectionally regulated anxiety and bone loss in mice. Specifically, activation of the Gi pathway prevented stress-induced anxiety in mice, while activation of the Gq pathway induced anxiety-like behavior in normal mice. After 4 weeks of intraperitoneal administration of CNO, chemogenetic regulation also had distinctive effects on BMD: notably, activation of the Gi and Gq pathways promoted and reduced BMD, respectively. Both hM3Dq and hM4Di can also induce intracellular calcium events in astrocytes [65]. These results suggest that astrocytes are functionally heterogenous in different brain regions [60, 66], and chemogenetic manipulation of astrocytes in different brain areas can cause complicated effects on cell activity.

### 3.2. VMH Astrocytes Regulate Excitability of SF-1 Neurons through Glutamate Receptors

In recent years, interactions between astrocytes and neurons have become a research hotspot [67]. Abnormal astrocytes can lead to neuronal dysfunction and the subsequent occurrence of various diseases. For example, astrocyte abnormalities can induce a variety of central nervous system diseases, including stroke, Alzheimer's disease, Parkinson's disease, Huntington's disease, and schizophrenia [68]. Furthermore, astrocytes not only provide energy support to neurons but also participate in the formation processing and pruning of synapses of neurons [69]. Moreover, astrocytes secrete a variety of gliotransmitters to regulate neuronal function and participate in extracellular neurotransmitter feedback in neurons to ensure the efficiency of information transmission. Astrocytes are known to secrete thrombospondin, which drives the formation of intact glutamatergic synapses by calcium channel regulation, thereby promoting the formation of structural synapses [70].

In this study, we found that selective regulation of astrocytes in the VMH bidirectionally regulated animal anxiety and bone loss. The most likely mechanism is that astrocyte activity changed the excitability of neurons, then regulated the relevant neural circuits, and finally exerted effects on animal anxiety and bone metabolism. To verify this hypothesis, we conducted electrophysiological recordings and found that optogenetically activated astrocytes induced SF-1 neuronal excitability by regulating NMDA receptors but not AMPA receptors, consistent with previous study on neurons cocultured with astrocytes [71]. NMDA receptors are obligatory heteromeric assemblies of two glycine-binding NR1 subunits and two other subunits (NR2A-D: glutamate-binding; NR3A-B: glycine-binding) [72]. Here, application of D-APV blocked the glutamate-binding site and significantly suppressed neuronal firing caused by astrocyte optogenetic activation, suggesting that glutamate-binding subunits are involved in this depolarization. Moreover, blockage of AMPA receptors did not suppress neuronal depolarization at the resting state induced by light illumination of astrocytes, indicating that Mg2 + -resistant NR2 subunits (like NR2C/D) are likely involved in this process as Mg2 + -sensitive subunits are blocked by Mg2+ at resting potentials [73]. However, our existing evidence could not determine the upstream mechanism underlying astrocyte regulation of anxiety-induced bone loss. For example, what kind of stimulation do these astrocytes receive in the state of anxiety, and what leads to the increase in their own activity and effects on SF-1 neuronal activity? These questions will be the focus of our future work.

### 3.3. Potential Mechanism of VMH Astrocytes in Regulating Anxiety-Induced Bone Loss

Our data support that VMH astrocytes regulate SF-1 neuronal activity through the NMDA receptor, which regulates anxiety and bone loss induced by chronic stress. However, the underlying mechanism by which chronic stress changes the function of astrocytes remains unclear. We previously showed that the concentration of VMH GABA is significantly higher in stressed mice than in control mice [26]. Whether astrocytes release glutamate under physiological conditions is controversial; however, recent studies have shown that GABA-induced upregulation of astrocyte calcium promotes the release of glutamate and ATP, and short-term high-concentration GABA stimulation induces astrocytes to release glutamate, which causes an excitatory response in neurons [44]. On the other hand, when astrocytes are stimulated with high concentrations of GABA long-term, glutamate and ATP are released to regulate the balance between excitability and inhibition of adnexal neurons [44, 74–77]. Therefore, high concentrations of GABA in the VMH brain area of chronically stressed mice could induce astrocytes to release glutamate and ATP to coordinate the function of SF-1 neurons and neural circuits by acting on different receptors, thereby regulating animal behavior and bone metabolism [28, 43, 78].

We also found that somatostatin- (SOM-) positive neurons in the BNST nucleus emit nerve projections to the astrocyte body of the VMH (data not shown). SOM neurons are reported to regulate intracellular calcium release of astrocytes by secreting GABA and SOM, thus regulating astrocyte activity [79]. These results suggest that BNST-VMH is not only composed of SOM and SF-1 neurons but also astrocytes from the VMH.

In conclusion, this study demonstrated that astrocytes in the VMH play an important role in regulating chronic stress-induced anxiety and bone loss. Optogenetic activation of VMH astrocytes induced an excitatory response in SF-1 neurons, which was mediated by NMDA receptors (Figure 5(g)). This study is the first to prove that VMH astrocytes not only participate in the regulation of emotional disorders but also regulate the process of bone metabolism. This expands our understanding of the function of astrocytes and provides a potential clinical intervention target for chronic stress-induced bone loss.

## 4. Materials and Methods

### 4.1. Animals

The animal experiments in this study were reviewed and approved by the Animal Ethics Committee of the Shenzhen Institute of Advanced Technology, Chinese Academy of Sciences. All animals used in this study were male. All bone analyses were performed on six-month-old mice, whereas electrophysiological recordings were performed on two-month-old mice. The S100*β*-Cre mice were purchased from KANGWEIDA Gene Technology Co., Ltd, Wuhan, China. Adult (6 weeks old) male C57BL/6 mice were purchased from the Guangdong Medical Laboratory Animal Center, Guangzhou, China. Animals were housed at 22–25°C on a 12 h : 12 h light-dark circadian cycle (lights on from 07 : 00 am to 19 : 00 pm) with food and water provided *ad libitum*. The animals were randomly allocated to experimental and control groups, and experimenters were blind to the experimental group during behavioral experiments. Experimental data were collected during the daytime period.

### 4.2. Unpredictable Chronic Mild Stress Procedure (UCMS)

The UCMS protocol was performed as described previously, with minor modifications [26]. The C57BL/6 or S100*β*-Cre mice were exposed to environmental stressors for 8 weeks, including the following: (i) tight squeeze: four mice were housed in a relatively small box (3 × 5 × 7 cm) for 2 h; (ii) wet environment: water was added to home cages to dampen bedding without generating large pools for 6 h; and (iii) high position stress: mice were placed on a platform raised 100 cm above floor height for 2 h. The stressors were randomized and counterbalanced so that each mouse received the same number of each stressor across consecutive days over 8 weeks. Efficacy of the induced anxiety procedures was confirmed by animal behavior studies.

### 4.3. Virus Injection and Manipulation

The viral vectors used in this study included AAV5-EF1a-DIO-hM4Di-mCherry, AAV5-EF1a-DIO-hM3Dq-mCherry, AAV5-EF1a-DIO-mCherry, AAV2-GFAP-ChR2-mCherry, and AAV2-GFAP-mCherry. Mice were fixed in a stereotaxic apparatus (RWD, China) once general anesthesia was achieved by pentobarbital sodium (intraperitoneal injection, 100 mg/kg). During surgery, the mice were anesthetized with isoflurane (1%) and kept warm with an electric blanket. A small hole in the skull above the targeted area was made with a dental drill, and injections were performed using a microsyringe pump (UMP3/Micro4, USA) with a 10 *μ*l syringe connected to a 33G needle (Neuros; Hamilton, Reno, USA) at a slow flow rate of 50 nl/min. To specifically infect astrocytes in the VMH with hM4Di-mCherry, hM3Dq-mCherry, or mCherry (control group) for DREADD manipulation, AAV5-EF1a-DIO-hM4Di-mCherry, AAV5-EF1a-DIO-hM3Dq-mCherry, or AAV5-EF1a-DIO-mCherry was injected into the VMH of S100*β*-Cre mice (virus titers: 1 × 10^13^ gc/ml, 0.3 *μ*l/injection; AP = −1.58 mm; ML = ±0.3 mm; DV = −5.35 mm); the coordinates of injection site were determined with the guide of brain atlas [80]. To specifically infect astrocytes in the VMH with ChR2-mCherry or mCherry for electrophysiological recordings, AAV2-GFAP-ChR2-mCherry or AAV2-GFAP-mCherry was injected into the VMH of C57BL/6 mice (virus titers: 1 × 10^13^ gc/ml, 0.3 *μ*l/injection; AP = −1.58 mm; ML = ±0.3 mm; DV = −5.35 mm). The volume and injection site of AAV virus were designed to ensure expression mainly constrained in VMH; data acquired from mice without expression of AAV in VMH had been excluded. After injection, mice infected with AAV vector were housed in specific-pathogen-free area to prevent possible infection.

When the injection was complete, the needle was left in place for 10 min and then extracted slowly to avoid virus leakage in the track. The wound was sutured, and antibiotics (bacitracin and neomycin) were applied to the surgical wound, with ketoprofen (5 mg/kg) injected subcutaneously. The animals recovered from anesthesia under a heat lamp before being returned to their cages. Mice were housed for 8–9 weeks following injection for viral expression concurrent with daily stress. Clozapine N-oxide (CNO) (1 mg/kg, Sigma, USA, C0832) was dissolved in 0.6% dimethyl sulfoxide (DMSO) in saline solution and delivered by intraperitoneal injection. For optogenetics manipulation, blue light (constant, 30 s) was delivered by a Lambda DG-4 (Sutter, USA) system under the control of Digidata 1440A (Molecular Devices, USA).

### 4.4. Immunostaining and Hematoxylin and Eosin (H&E) Staining

Brains were perfused with and fixed in 4% paraformaldehyde (PFA) at 4°C overnight and cryosectioned at a thickness of 30 *μ*m. Sections were then rehydrated and blocked by goat serum. The sections were incubated with primary antibodies to GFAP (1 : 1000, Abcam, ab7260 or ab10062, UK) and anti-S100*β* (1 : 500, Abcam, ab868, UK). The sections were then washed and labeled with fluorescence-conjugated corresponding secondary antibodies (Jackson ImmunoResearch, 111-545-003, 315-545-003, 1 : 500, USA). The sections were counterstained with Hoechst 33342 and then mounted for image acquisition. Images were taken under a microscope (LSM880, Zeiss, Germany). The numbers of c-fos-positive cells or c-fos/GFAP colocated in the VMH were counted in four sections (adjacent levels) from each mouse, with each group containing 5 mice. The c-fos expression is considered an indirect marker of recent astrocyte activity, and many studies have shown that c-fos is expressed in astrocytes in response to various extracellular signals [81, 82]. The cell numbers per section were compared among experimental groups during data analyses. The signal intensities and fiber length of GFAP staining were quantified by the ZEN software (Zeiss, Germany). H&E staining was performed as described previously [23].

### 4.5. Slice Preparation

Mice were deeply anesthetized with isoflurane and decapitated rapidly. Brains were then removed and placed to cold oxygenated cutting solution (95% O_2_ and 5% CO_2_) with the following composition (in mM): choline chloride 110, KCl 2.5, Na-pyruvate 0.6, MgCl_2_ 7.0, CaCl_2_ 0.5, NaH_2_PO_4_ 1.3, NaHCO_3_ 25, and glucose 20 (pH 7.4). Coronal slices (250–300 mm thick) were cut with a vibratome (Series 1000, Warner Instruments, Berlin, Germany) and incubated at 34°C for 30 min in oxygenated artificial cerebrospinal fluid (aCSF, in mM): NaCl 125, KCl 2.5, Na-pyruvate 0.6, MgCl_2_ 1.3, CaCl_2_ 2.0, NaH_2_PO_4_ 1.3, NaHCO_3_ 25, and glucose 10 (pH 7.4). After incubation, all slices were equilibrated in aCSF at room temperature (24–26°C) for at least 40 min. Single slices were transferred to the recording chamber perfused with oxygenated aCSF at room temperature. Unless stated otherwise, drugs were applied with perfused aCSF.

### 4.6. Electrophysiology

All VMH neurons were recorded with whole-cell patch clamp recording configuration. Recordings were obtained with multiclamp 700B amplifiers (Molecular Devices, San Jose, USA) under visual guidance using a Nikon FN1 microscope (Tokyo, Japan). Electrophysiological data were acquired and analyzed using the pClamp 10 software (Molecular Devices, San Jose, USA). Whole-cell recording pipettes were pulled (model PC-100, Narishige, Tokyo, Japan) from borosilicate glass (0.69 mm OD, 5–7 M*Ω*) and filled with solution containing (in mM): K-gluconate 135.0, KCl 4.0, NaCl 2.0, HEPES 10, EGTA 4.0, Mg-ATP 4.0, and Na-GTP 5.0. Osmolality was adjusted to 290–310 mOsm kg^−1^ with sucrose, and pH was adjusted to 7.4 with KOH. The liquid junctional potential was compensated for by using the “Pipette Offset” setting of the multiclamp 700B amplifier when the pipette contacted the bath solution.

After forming a high-resistance seal (G*Ω*), the cell was held in current-clamp mode for 7–10 min until access resistance stabilized. To elucidate how astrocytes in the VMH affect the electrophysiology of neurons, we applied blue light illumination with the Lambda DG-4 system (Sutter, Novato, USA) to activate ChR2-expressing astrocytes. NBQX (50 *μ*M, MCE, Shanghai, China) and D-APV (50 *μ*M, MCE Shanghai, China) were used in perfused aCSF to block the function of AMPA and NMDA receptors, respectively.

### 4.7. Animal Behavior Studies

Littermate mice were randomly allocated to experimental and control groups. Experimenters were blind to the experimental group allocation. To reduce experimenter-introduced interference, all mice were handled for 15–30 min per day for 3 d before behavioral tests. In this study, the open-field and elevated plus maze tests were employed for measuring anxiety in mice.

### 4.8. Open-Field Test

Anxiety level was measured in a white plastic open-field square chamber (50 cm length × 50 cm width × 40 cm height). Briefly, the mice were placed in a random corner of the arena at the start of each test and allowed to explore for 10 min. All animal activity was recorded with an infrared camera placed above the box. The arena was conceptually divided into a central field (25 × 25 cm) and a peripheral field. The number of entries into and time spent in the central area during the 10 min of exploration was measured (ANY-maze software). The chamber was cleaned with 30% alcohol and dried thoroughly after each test session to remove any odor cues left by the former mice.

### 4.9. Elevated Plus Maze Test

We also used the elevated plus maze apparatus to measure anxiety, which entailed a plastic maze consisting of a central platform (5 × 5 cm) with two white open arms (25 × 5 × 25 cm) and two white closed arms (25 × 5 × 25 cm) extending from the center in a plus shape. The maze was elevated 65 cm above the floor. The mice were individually placed in the center with their heads facing a closed arm and were allowed to explore the maze for 5 min. A high-definition camera was set above the apparatus to record animal activity. The number of entries into and amount of time spent in each arm was recorded (ANY-maze software). The maze was cleaned with 30% alcohol and dried thoroughly after each test session to remove any odor cues left by the former mice.

### 4.10. Bone Mineral Density (BMD) Measurement

The perfused or separated leg bones were placed in a small animal body composition analyzer (InAlyzer, South Korea), and BMD was determined using a dual-energy X-ray analysis system. The samples were placed in the same horizontal plane and direction for dual-energy X-ray scanning; after scanning, the area of interest (5 × 5 mm) was uniformly circled to calculate the BMD of the samples, and the differences in BMD in each group were statistically analyzed by the GraphPad Prism (GraphPad Software, USA) software.

### 4.11. Statistics

All statistics were performed using the GraphPad Prism 8.0 software. Unless otherwise specified, when appropriate, paired Student*t-*tests and unpaired Student*t-*tests were used. Bonferroni *post hoc* comparisons were conducted to detect significant effects or interactions. In all statistical indicators, *p* < 0.05 was statistically significant. Unless stated otherwise, values represent mean ± standard deviation (SD).

## Data Availability

The datasets used and analyzed in the current study are available from the corresponding author upon reasonable request.
